# Bacterial Membrane Vesicles as Versatile Platforms for Systemic and Mucosal Vaccines

**DOI:** 10.3390/vaccines14050440

**Published:** 2026-05-15

**Authors:** Si Hyun Park, Young Min Son

**Affiliations:** Department of Systems Biotechnology, Chung-Ang University, Anseong 17546, Republic of Korea

**Keywords:** vaccine platform, bacterial membrane vesicles, self-adjuvanting system, systemic immunity, mucosal immunity

## Abstract

Bacterial membrane vesicles (BMVs), encompassing outer membrane vesicles (OMVs) released from Gram-negative bacteria and extracellular vesicles (EVs) released from Gram-positive bacteria, have emerged as promising vaccine platforms owing to their intrinsic immunostimulatory properties and capacity to deliver a wide range of antigens. Although conventional vaccines effectively prevent infectious diseases, their long-term efficacy is often limited by antigenic variation and reliance on a restricted number of licensed adjuvants. BMVs, as self-adjuvanting systems, enable both antigen delivery and innate immune activation. BMVs are nanoscale lipid bilayer structures enriched with pathogen-associated molecular patterns (PAMPs), facilitating their recognition and uptake by antigen-presenting cells. This leads to the activation of pattern recognition receptors and the induction of pro-inflammatory cytokines, type I interferons, and adaptive immune responses, including antibody production and Th1- and Th17-biased cellular immunity. Recent studies highlight the versatility of BMVs as vaccine platforms across bacterial, fungal, and viral infection models. BMVs induce protective immunity by promoting both systemic and mucosal immune responses, thereby reducing bacterial burden and limiting pathogen colonization across diverse infection models. These properties have supported their application in viral vaccine development, including influenza and SARS-CoV-2, with the potential to enhance mucosal immunity. Despite these advantages, challenges remain in standardization, safety, and antigen-loading efficiency. Engineered BMVs incorporating protein or mRNA antigens may further enhance antigen presentation and CD8^+^ T cell responses. This review summarizes the biological features, immunological mechanisms, and future potential of BMVs in vaccine development.

## 1. Introduction

Vaccination has long been recognized as one of the most effective public health strategies for preventing infections caused by viruses and pathogenic microorganisms [1,2]. Particularly, the global experience with the SARS-CoV-2 pandemic, along with recurrent outbreaks of respiratory viruses such as influenza, has further heightened both societal and scientific awareness of the critical importance and necessity of effective vaccine strategies [3]. However, the rapid antigenic variation in viruses can limit the long-term effectiveness of existing vaccines [4,5]. Moreover, current vaccine formulations often depend on adjuvants, yet only a limited number of adjuvants are licensed for human use, and many require complex manufacturing processes that increase production costs [6,7]. In this regard, the development of vaccine platforms with high immunogenicity and broad applicability against diverse pathogens has become an increasingly important research priority.

To address these challenges, a variety of next-generation vaccine platforms have been actively developed, including mRNA vaccines, viral vector vaccines, recombinant subunit vaccines, and synthetic nanoparticle-based platforms such as virus-like particles (VLPs) and lipid nanoparticles (LNPs) [8]. While each platform offers distinct advantages, they also exhibit intrinsic limitations in immunogenicity, adjuvant dependency, mucosal immunity, manufacturing complexity, or storage stability. For example, mRNA-LNP vaccines achieve rapid antigen production and strong systemic immunity but require ultra-cold-chain storage and provide limited mucosal protection [9,10]. Viral vector vaccines induce robust cellular and humoral responses, yet their efficacy can be compromised by pre-existing or vector-induced anti-vector immunity [11]. Recombinant subunit and VLP vaccines offer favorable safety profiles but rely on co-administered adjuvants, only a few of which are clinically licensed for human use [6,7]. In this context, bacterial membrane vesicles (BMVs) have emerged as a uniquely positioned platform that intrinsically integrates antigen delivery and innate immune activation, occupying a complementary niche among existing vaccine technologies.

As a representative example of such a complementary platform, bacterial membrane vesicles (BMVs) secreted by various microorganisms, particularly bacteria, have been explored as promising vaccine candidates. BMVs encompass two major subtypes: outer membrane vesicles (OMVs), which are released from the outer membrane of Gram-negative bacteria and characteristically contain lipopolysaccharide and outer membrane proteins, and extracellular vesicles (EVs), which originate from the cytoplasmic membrane of Gram-positive bacteria [12,13]. Inherently derived from bacterial membranes, BMVs harbor a diverse array of antigens both on their surface and within their internal lumen, while simultaneously incorporating multiple pathogen-associated molecular patterns (PAMPs) [14]. Consequently, BMVs serve as both efficient antigen delivery vehicles and self-adjuvanting platforms that activate the innate immune system without the need for exogenous adjuvant treatment.

Several studies have demonstrated that BMVs are efficiently recognized and internalized by dendritic cells and macrophages, where they promote antigen presentation and drive a range of adaptive immune responses, including Th1- and Th17-skewed T cell responses as well as durable antibody production [15,16,17]. Based on these immunological properties, BMVs exhibit strong potential not only as vaccines against bacterial infections but also as versatile platforms applicable to viral and fungal vaccination [18,19,20]. Recent studies have explored a broad spectrum of strategies, including the direct use of native BMVs as vaccines, the development of engineered vesicles designed to display or encapsulate defined antigens, and mucosal vaccination approaches aimed at enhancing respiratory immunity [21,22,23].

In this review, we summarize the concept and biological characteristics of bacterial membrane vesicles (BMVs) and discuss the immunological mechanisms through which these vesicles activate the host immune system. We then highlight recent advances in vaccine development using BMVs derived from Gram-negative and Gram-positive bacteria, as well as their extension to fungal and viral vaccine platforms. Finally, we discuss the advantages and limitations of BMV-based vaccines and consider their potential as next-generation vaccination technologies.

## 2. Overview of BMVs

BMVs are nanoscale membrane structures released from bacteria that participate in diverse biological processes, including intercellular communication, delivery of virulence factors, and modulation of host immune responses [24]. Owing to these intrinsic biological functions and their modifiable nature, BMVs have emerged as promising platforms for therapeutic and vaccine applications.

### 2.1. Definition and Classification of BMVs

BMVs are lipid membrane-based nanoscale vesicles that are naturally released from bacteria under physiological or pathological conditions and are defined as structures that encapsulate diverse biomolecules, including proteins, lipids, and nucleic acids [13,14].

As briefly introduced above, in Gram-negative bacteria, the released vesicles are typically referred to as OMVs, as they originate from the outer membrane and characteristically contain outer membrane proteins and lipopolysaccharide (LPS) [25].

In contrast, in Gram-positive bacteria, which possess a thick peptidoglycan cell wall, the release of membrane-derived EVs has been reported. These Gram-positive EVs originate from the cytoplasmic membrane and typically contain lipoproteins, lipoteichoic acid(LTA), peptidoglycan-associated molecules, toxins, and other virulence factors, but lack the LPS that characterizes Gram-negative OMVs [26].

Ongoing efforts to classify BMVs based on their biogenesis, size, molecular composition, and functional characteristics not only advance our understanding of their biological roles but also provide an essential foundation for assessing their potential as vaccine and therapeutic platforms [27].

### 2.2. BMV Biogenesis, Structural Characteristics, and Distribution of BMVs

BMVs are formed through localized deformation of the bacterial membrane, followed by vesicle budding and release. In Gram-negative bacteria, OMV biogenesis has been explained by several models. The classical model proposes that OMVs are generated when localized loss of crosslinks between the outer membrane and the underlying peptidoglycan layer—often associated with reduced anchoring by lipoproteins such as Lpp and OmpA—leads to outward bulging and pinching off of the outer membrane [28]. A second model emphasizes asymmetric accumulation of LPS and phospholipids within the outer membrane, in which dysfunction of the VacJ/Yrb lipid transport system promotes membrane curvature that favors vesiculation [29]. A third model relates OMV release to envelope stress responses, in which the accumulation of misfolded proteins or peptidoglycan fragments within the periplasm enhances vesicle formation [30]. Additionally, certain bacteria such as *Neisseria gonorrhoeae*, *Pseudomonas aeruginosa*, and *Acinetobacter baumannii* have been reported to release outer–inner membrane vesicles (O-IMVs), which contain components from both the outer and inner membranes, along with cytoplasmic contents [31].

In contrast, Gram-positive bacteria, which possess a thick peptidoglycan cell wall, have been shown to release Gram-positive EVs through specialized mechanisms that allow vesicles to pass through the thick cell wall. A representative mechanism is “bubbling cell death,” in which prophage-encoded endolysins locally weaken the peptidoglycan layer, allowing cytoplasmic membrane protrusions to bulge outward and release as vesicles [32]. As a complementary mechanism, peptidoglycan turnover and remodeling by autolysins generate transient pores or microchannels within the cell wall, providing additional routes through which vesicles can be released [26].

Structurally, BMVs are spherical nanoscale vesicles surrounded by a lipid bilayer, typically ranging from approximately 50 to 200 nm in diameter. Their surfaces display membrane-derived lipids and proteins, such as lipopolysaccharides, lipoproteins, and outer membrane proteins, whereas the lumen contains a wide variety of bacterial components, including toxins, enzymes, metabolites, and nucleic acids [33,34]. These structural and molecular features provide a robust basis for BMVs to function as both stable antigen carriers and potent immune stimulators, thereby supporting their potential as promising platforms for vaccine development.

For BMVs to be developed as therapeutic and vaccine platforms, understanding their in vivo distribution within the host is essential. Beyond in vitro studies, BMVs have been detected as biologically active structures across diverse body fluids, including blood, urine, saliva, and bronchoalveolar lavage fluid, reflecting their continuous release by commensal and pathogenic bacteria. Microbiota-derived BEVs have been identified in the blood of healthy donors [35], and gut-derived OMVs can translocate across the intestinal epithelium into the systemic circulation, reaching distal organs such as the liver in murine models [36]. In contrast, the distribution of vaccine-administered BMVs is largely shaped by the route of immunization: following intramuscular or subcutaneous delivery, BMVs primarily drain to local lymph nodes where they are taken up by antigen-presenting cells, with a fraction entering the systemic circulation and reaching the spleen and liver [37]. Thus, while BMVs naturally circulate as measurable entities within host body fluids, vaccine-administered BMVs are preferentially routed to draining lymph nodes upon immunization, where concentrated APC uptake underlies their immunogenic efficacy as a vaccine platform.

### 2.3. Molecular Cargo and Immune Recognition of BMVs

**Figure 1:**
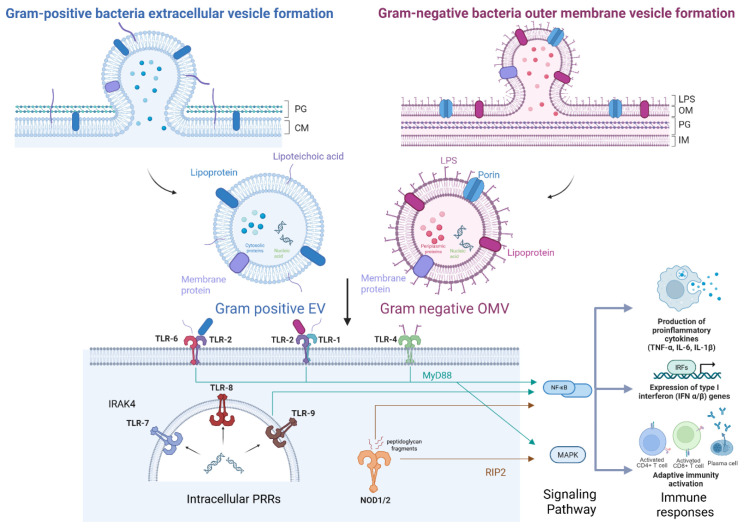
Biogenesis of bacterial membrane vesicles (BMVs) and their immunostimulatory pathways. Gram-positive bacteria release extracellular vesicles (EVs) through cytoplasmic membrane budding across the peptidoglycan layer, whereas Gram-negative bacteria generate outer membrane vesicles (OMVs) from the outer membrane containing lipopolysaccharide (LPS) and periplasmic components. These vesicles are recognized by surface and intracellular PRRs, including TLRs and NOD1/2, activating MyD88-, IRAK4-, and RIP2-dependent NF-κB and MAPK signaling. This leads to pro-inflammatory cytokine production, type I interferon responses, and activation of adaptive immunity. Created with BioRender.com.

BMVs are membrane-derived structures originating from bacterial membranes and contain a diverse range of molecular cargo that provides important immunological cues enabling their recognition by the host immune system (Figure 1). Particularly, BMVs are enriched with PAMPs, including LPS, lipoproteins, flagellin, and peptidoglycan fragments [38]. These PAMPs exist in diverse molecular forms, including lipids, proteins, carbohydrates, and nucleic acids, and are recognized by various pattern recognition receptors (PRRs) expressed on host immune cells [39,40]. Engagement of these receptors triggers downstream signaling cascades, including activation of the NF-κB and MAPK pathways, induction of inflammasome signaling, and, in certain contexts, type I interferon responses [41,42,43].

Representative PAMPs present in Gram-negative OMVs include LPS, which is recognized by the TLR4–MD-2 complex. This interaction activates MyD88-dependent signaling pathways and subsequently induces NF-κB and MAPK signaling cascades. These signaling events ultimately promote the production of pro-inflammatory cytokines such as TNF-α, IL-6, and IL-1β [14,42,44,45,46]. On the other hand, Gram-positive EVs predominantly carry lipoproteins, lipopeptide components, and lipoteichoic acid (LTA), which are recognized by TLR2 in cooperation with TLR1 or TLR6, forming functional heterodimers. This recognition triggers MyD88-dependent signaling pathways, leading to the induction of proinflammatory cytokines and chemokines [47]. Notably, TLR2-mediated signaling provides broad recognition of bacterial membrane-derived components, allowing innate immune responses to be elicited by membrane vesicles from a wide range of bacterial species [46,48,49].

Furthermore, BMVs carry various nucleic acids that are sensed by endosomal Toll-like receptors TLR7, TLR8, and TLR9 after vesicle internalization by antigen-presenting cells [50]. Engagement of TLR7/8/9 within endosomal compartments activates MyD88-dependent signaling pathways, leading to the activation of NF-κB and IRF7. As a result, these signaling events promote the production of type I interferons and interferon-stimulated genes, thereby driving antiviral innate immune responses that are distinct from the proinflammatory cytokine responses primarily induced by surface TLR signaling [51,52].

Beyond recognition mediated by Toll-like receptors, PAMPs contained within BMVs can also be sensed by cytosolic PRRs. Among these, the nucleotide-binding oligomerization domain (NOD) receptors NOD1 and NOD2 are representative cytosolic PRRs that recognize bacterial peptidoglycan-derived components and play important roles in innate immune responses elicited following entry of BMVs into host cells [53,54]. Upon internalization, BMVs can be degraded within the cytosol or expose their molecular cargo, allowing detection by NOD1 or NOD2. This sensing event activates RIP2-dependent signaling pathways, leading to the activation of NF-κB and MAPK signaling cascades. Consequently, these pathways drive the expression of proinflammatory cytokines and chemokines, thereby contributing to the initiation and amplification of innate immune responses against BMVs [55,56].

### 2.4. BMV–Host Immune Interactions: From Innate Recognition to Adaptive Protection

The PRR-mediated innate signaling discussed in Section 2.3 provides the basis for understanding how BMV-based vaccines shape adaptive immunity. Depending on their bacterial origin and vesicle composition, BMVs contain multiple PAMPs that activate antigen-presenting cells and induce cytokines such as IL-12, IL-6, and IL-23, thereby supporting Th1- and Th17-biased responses [57,58]. Depending on the route of immunization and source bacteria, BMV immunization can also induce Th2-associated responses, as shown by IL-4, IL-10, IL-13, and IgG1 production following oral immunization with *H. pylori*-derived OMVs [59]. In parallel, the particulate structure of BMVs presents repeated antigens together with PAMPs, enabling BCR–TLR cooperation that supports B cell activation and antibody class switching [60,61]. Thus, BMV-induced protection should be viewed as an adaptive immune outcome shaped by APC activation, T helper polarization, and B cell responses, rather than as a simple consequence of innate immune stimulation.

CD4^+^ T-cell polarization is a key determinant of BMV-induced protection, and can be biased toward Th1, Th17, or Th2 depending on vesicle origin and route of administration. A representative Gram-positive EV model illustrates Th1-type cellular immunity: in *S. aureus* EV-immunized mice, splenic T cell transfer reproduced protection whereas immune serum transfer did not, and protection was markedly reduced in IFN-γ^−^/^−^ mice but only minimally affected in IL-17^−^/^−^ mice, identifying Th1-derived IFN-γ as the dominant effector mechanism [15]. Some OMVs induce Th17-biased responses with mucosal relevance: *P. gingivalis* OMVs promoted DC-derived IL-6 and IL-23 production, supporting Th17 differentiation [62], whereas intranasal *B. pertussis* OMVs elicited Th17 responses associated with IL-17-mediated neutrophil recruitment and airway colonization control [63]. In contrast, oral *H. pylori* OMVs induced Th2-associated cytokines, including IL-4, IL-10, and IL-13, together with IgG1 production, suggesting a contribution of Th2-biased humoral immunity to protection [59]. Thus, BMV-induced T-cell immunity is highly context-dependent and distributed across a Th1/Th17/Th2 spectrum shaped by vesicle composition, route of administration, and the APC-derived cytokine milieu.

B cell-mediated humoral immunity also represents an important axis of BMV-induced protection. Following systemic immunization, BMV-associated antigens activate antigen-specific B cells in draining lymph nodes, generating antigen-experienced B cell populations that, with T follicular helper (Tfh) cell help, undergo germinal center reactions, class switching, and differentiation into plasmablasts and antibody-secreting plasma cells [64]. The resulting antigen-specific IgG contributes to systemic protection by neutralizing pathogens, promoting opsonophagocytosis, and activating complement, thereby limiting systemic dissemination and bacterial burden [65,66]. In contrast, mucosal immunization induces IgA class switching within NALT and GALT, generating IgA-secreting plasma cells whose dimeric IgA is transported across the epithelium by the polymeric immunoglobulin receptor and released as secretory IgA, which limits pathogen adhesion, colonization, and epithelial invasion [67,68,69]. Thus, BMV-induced humoral immunity is functionally compartmentalized into systemic IgG-mediated and mucosal sIgA-mediated protection, shaped primarily by route of administration and vesicle antigen composition.

Building on the above, BMV-induced protective immunity emerges as a multi-axis adaptive outcome, in which antigen-experienced populations can give rise to long-lived plasma cells, memory B cells, and tissue-resident memory T cells, supporting durable immune memory. In line with this view, immunization of mice with *B. pertussis*-derived OMVs led to detection of lung-resident memory CD4 T cells (T_RM_), confirming the establishment of cellular memory [70], whereas intranasal administration of the same OMVs generated lung-resident IgA memory B cells and IgA-/IgG-producing plasma cells in the lung together with systemic memory B cells in the spleen, indicating that humoral memory was induced at both mucosal and systemic sites [60]. Although it is well established in general immunology that TLR signaling contributes to adaptive immune cell activation, mechanistic studies that directly demonstrate how specific TLR signals drive defined Th lineages or B cell subsets in the BMV context remain very limited. In the case of *S. aureus* EVs, the induction of IL-12 by APCs and the requirement for Th1-derived IFN-γ as the dominant effector cytokine were demonstrated [15], suggesting that TLR2/MyD88 signaling may contribute to Th1 polarization through APC activation; however, direct receptor-level validation of this connection is still lacking. Therefore, longitudinal analysis of BMV-induced cellular and humoral memory, together with receptor-level mechanistic dissection of PRR–adaptive lineage connections, remains a central challenge for the BMV vaccine field.

## 3. Direct BMV Vaccines Against Infectious Disease Models

### 3.1. Bacterial Infection

Antibiotic therapy has long been the primary strategy for the treatment of bacterial infections; however, the widespread emergence of antibiotic-resistant strains has made it increasingly difficult to effectively control infections using this approach alone [71,72]. To address these limitations, vaccine-based strategies that harness host immune responses to prevent infection or mitigate disease severity are emerging as important complementary approaches [73,74]. In this context, BMVs have emerged as promising vaccine platforms, as they simultaneously incorporate diverse bacterial antigens and pathogen-associated molecular patterns, enabling the induction of both systemic and mucosal immune responses.

#### 3.1.1. Gram-Negative Bacteria-Derived OMVs

**Figure 2:**
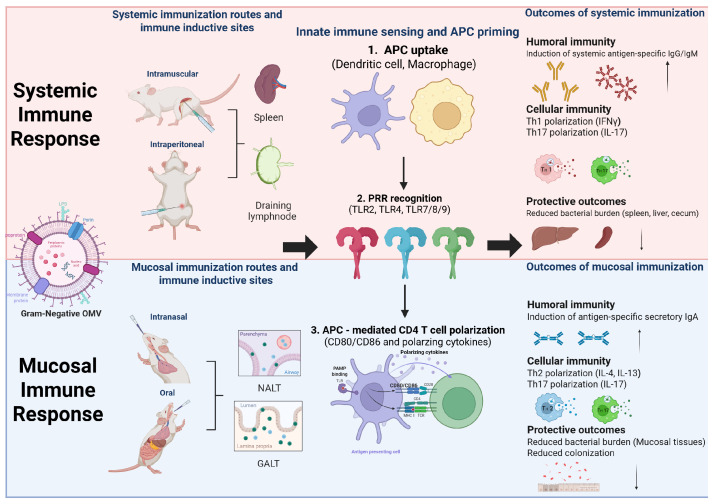
Integrated immune signal axis of OMV-induced systemic and mucosal immunity. Gram-negative outer membrane vesicles (OMVs) contain multiple PAMPs and act as self-adjuvanting vesicles that connect innate sensing with adaptive immunity. Systemic immunization via intramuscular or intraperitoneal routes engages draining lymph nodes and the spleen, whereas intranasal or oral immunization engages mucosal inductive sites, including NALT and GALT. OMVs are taken up by APCs and recognized by PRRs, leading to CD80/CD86 upregulation and polarizing cytokine production. These signals induce systemic IgG/IgM and Th1/Th17 responses after systemic immunization, or secretory IgA and Th17 or Th2-associated responses after mucosal immunization, thereby reducing bacterial burden, inflammation, and colonization. Created with BioRender.com.

Gram-negative bacteria are characterized by a distinctive cell envelope architecture consisting of a thin peptidoglycan layer surrounded by an outer membrane. This outer membrane contains a variety of pathogen-associated molecular patterns, including LPS, which are capable of eliciting host innate immune responses [75,76]. OMVs, which are naturally released from this outer membrane, simultaneously incorporate bacterial antigens and immunostimulatory signals derived from the source organism. These properties provide a strong biological basis for the recognition of Gram-negative bacteria-derived OMVs as vaccine platforms that integrate antigen delivery with innate immune activation (Figure 2).

Systemic vaccination strategies employing OMVs derived from Gram-negative bacteria have been consistently reported to induce protective immunity across diverse animal models. Particularly, murine studies have provided extensive evidence supporting the efficacy of Gram-negative bacteria-derived OMVs as systemic vaccine platforms. Intraperitoneal immunization with *Klebsiella pneumoniae*-derived OMVs in mice induced both antigen-specific antibody responses and T cell-mediated immunity, resulting in a significant increase in survival following lethal bacterial challenge. This protective effect was attributed to the coordinated contribution of humoral and cellular immune responses [77].

Similarly, intramuscular immunization with *A. baumannii*-derived OMVs elicited robust systemic immune responses, characterized by enhanced serum antibody production, improved survival after bacterial challenge, and a marked reduction in bacterial burden in systemic organs such as the spleen [66]. In a murine sepsis model, repeated intraperitoneal immunization of *Escherichia coli*-derived OMVs conferred protection against bacteria-induced lethality and systemic inflammatory responses. Mechanistically, OMV immunization promoted the production of Th1- and Th17-polarizing cytokines by dendritic cells and led to increased IFN-γ and IL-17 expression in CD4^+^ T cells, highlighting the importance of Th1·Th17-biased cellular immunity in mediating systemic protection against bacterial infection [16].

The efficacy of systemic OMV-based vaccines has also been demonstrated in avian models. In chicks, intramuscular immunization with a mixture of OMVs derived from avian pathogenic *E. coli* (APEC) O1, O2, and O78 strains induced robust systemic antibody responses and conferred protection against APEC infection [78]. Likewise, intramuscular vaccination with OMVs derived from *Salmonella enterica* serovar Enteritidis resulted in the generation of serum IgG and IgM antibodies and significantly reduced bacterial colonization in the liver, spleen, ileum, and cecum [79]. These findings underscore the potential of OMV-based systemic vaccines to effectively control major Gram-negative bacterial infections in poultry.

Collectively, these studies demonstrate that systemic immunization with Gram-negative OMVs—via intramuscular or intraperitoneal routes—induces coordinated antibody and Th1/Th17 responses, improving survival and reducing bacterial burdens across animal models. Beyond systemic immunity, OMV-based vaccines can also elicit localized mucosal responses following intranasal or oral immunization. The route of administration shapes the inductive site engaged: intranasal delivery activates the nasal-associated lymphoid tissue (NALT), whereas oral delivery engages the Peyer’s patches within the gut-associated lymphoid tissue (GALT) [80], where antigen-experienced B cells differentiate into IgA-secreting plasma cells, and dimeric IgA is transported across the epithelium by the polymeric immunoglobulin receptor to form secretory IgA (sIgA) [81,82]. Tissue-resident memory T cells (T_RM_) lodged within these tissues further support mucosal protection through rapid cytokine secretion upon antigen re-exposure [83]. Unlike parenteral vaccines that mainly elicit systemic IgG, mucosal immunization induces both sIgA and T_RM_, enabling protection before pathogen attachment and limiting onward transmission [84], thereby bridging systemic and mucosal immunity.

In a representative study, OMVs derived from *A. baumannii* were evaluated following intranasal immunization to investigate their capacity to induce mucosal immune responses. Intranasal immunization promoted the generation of antigen-specific IgA in nasal secretions and alleviated infection-associated body weight loss. Notably, bacterial burdens in the bronchoalveolar lavage fluid (BALF), nasal tissue, and lung tissue were significantly reduced following intranasal immunization, with these effects being greater than those observed after subcutaneous or intramuscular immunization. These findings indicate that intranasal delivery of Gram-negative OMVs can effectively suppress airway colonization and limit infection dissemination within the respiratory tract, highlighting the contribution of mucosal IgA-associated immunity to protective outcomes [69].

The relevance of OMV-based vaccines for respiratory bacterial infections has been further demonstrated in studies targeting Gram-negative pathogens that primarily infect the airway. In one such study, intranasal immunization with OMVs derived from *Bordetella pertussis* induced robust antigen-specific IgA responses within the lung and airway mucosa, accompanied by a marked increase in Th17 cell responses. These mucosal IgA- and Th17-associated immune responses were closely linked to broad protective efficacy against *B. pertussis* infection, highlighting the ability of OMV-based vaccines to establish effective local immune defenses within the respiratory mucosal environment [63].

Beyond the respiratory tract, OMV-based vaccines have also been investigated for their ability to induce protective immune responses within the gastrointestinal mucosa. The potential of OMV-based mucosal vaccines has also been demonstrated in a study targeting *Helicobacter pylori*, a pathogen that colonizes the gastric mucosa. In a mouse model, oral immunization with *H. pylori*-derived OMVs induced strong systemic antibody responses and elevated levels of secretory IgA in gastric tissues and mucosal secretions, exceeding those observed with conventional whole-cell vaccines. OMV immunization also promoted a Th2-biased immune response characterized by increased IgG1 production and elevated levels of IL-4, IL-10, and IL-13. These responses were associated with reduced bacterial burden and urease activity following infection challenge, demonstrating effective protection against *H. pylori* infection [59]. This Th2-biased outcome contrasts with the predominantly Th1/Th17-oriented responses observed with most BMV-based vaccines, illustrating that BMV-induced Th polarization is context-dependent and shaped by the source pathogen, delivery route, and tissue microenvironment.

Taken together, the mucosal immunization studies described above show that OMV-based vaccination can induce protective immune responses at mucosal surfaces. Intranasal immunization with OMVs derived from respiratory pathogens such as *A. baumannii* and *B. pertussis* promotes antigen-specific mucosal IgA responses and cellular immunity, including Th17-associated responses, leading to reduced bacterial colonization and enhanced protection in the respiratory tract. OMV-based vaccines have also shown the capacity to induce protective immunity within the gastrointestinal mucosa, as demonstrated in a study targeting *H. pylori*. These findings highlight the versatility of OMV platforms in eliciting effective mucosal immune responses across multiple barrier tissues.

#### 3.1.2. Gram-Positive Bacteria-Derived EVs

As discussed earlier (Section 2), Gram-positive bacteria lack an outer membrane and instead release EVs derived from the cytoplasmic membrane through the thick peptidoglycan cell wall [75]. Nevertheless, in contrast to Gram-negative OMV biogenesis, the mechanisms underlying EV release across the thick peptidoglycan layer in Gram-positive bacteria remain relatively poorly understood, and the factors controlling EV composition and secretion have yet to be fully defined [85]. Despite these structural and biological constraints, one potential advantage of Gram-positive EVs is the absence of LPS, a major endotoxin present in Gram-negative OMVs, which may reduce endotoxin-associated toxicity and provide a more favorable safety profile for vaccine applications. Consequently, Gram-positive EVs have increasingly been investigated as potential vaccine platforms [15,86,87,88].

Interestingly, a study using EVs derived from *Staphylococcus aureus* provided more definitive evidence for the protective potential of Gram-positive EV-based vaccines. *Staphylococcus aureus* EVs promoted strong innate immune activation by upregulating CD80 and CD86 expressions and inducing the secretion of TNF-α, IL-6, and IL-12 in dendritic cells. In murine models, repeated intramuscular immunization with EVs elicited robust antigen-specific IgG antibody responses, and the resulting protective immunity was mediated predominantly by Th1 cell responses, with partial contributions from Th17 cells. Notably, immunized mice exhibited 100% survival following lethal *S. aureus* challenge even 40 days after the final vaccination, demonstrating that Gram-positive EVs can induce functionally protective immunity in infection challenge models [15].

Further extension of the translational relevance of Gram-positive EV-based vaccines was demonstrated in studies using EVs derived from *Streptococcus pneumoniae*. In this study, mice immunized intramuscularly with EVs formulated with aluminum hydroxide adjuvant developed strong EV-specific IgG antibody responses and showed significantly improved survival following lethal pneumococcal challenge. Importantly, these EVs also exhibited high immunoreactivity with human sera, particularly sera obtained from patients with pneumococcal infection, indicating that the EVs effectively reflect antigens expressed during natural infection [87].

Nevertheless, in a study employing EVs derived from *Clostridium perfringens* type A strains, the EVs were shown to be efficiently internalized by macrophages and to induce robust immunostimulatory effects, characterized by the production of proinflammatory cytokines such as TNF-α, IL-6, and G-CSF under both in vitro and in vivo conditions. Repeated intraperitoneal immunization with EVs in mice effectively elicited serum IgG antibody responses; however, mucosal IgA responses remained limited, and the induced immunity was insufficient to confer complete protection against infection with the corresponding bacterial strain. These findings suggest that although Gram-positive EVs possess the ability to modulate the host immune environment, their protective efficacy as standalone vaccines may be limited [86].

Overall, vaccine studies utilizing EVs derived from Gram-positive bacteria have demonstrated meaningful progress in inducing antigen-specific antibody responses and Th1-dominant systemic cellular immunity. However, most successful examples reported to date rely on systemic immunization routes, such as intramuscular or intraperitoneal immunization, whereas reports of effective mucosal immunization remain limited. Intranasal *Streptococcus mutans* EVs with hydroxyapatite adjuvant induced mucosal IgA and systemic IgG responses [89], though such examples without adjuvants remain scarce. This contrasts with Gram-negative OMV-based vaccines, which have been shown to elicit both local mucosal and systemic immune responses following mucosal delivery, and highlights a key challenge that remains to be addressed in the development of Gram-positive EV-based vaccine platforms. Furthermore, these findings underscore that the immunogenicity and protective efficacy of Gram-positive EVs can vary significantly depending on the specific bacterial species and strain from which they are derived (Table 1).

### 3.2. Fungal Infection

Over the past two decades, the incidence of fungal infections has increased markedly, particularly among immunocompromised individuals and the elderly, driven by factors such as the widespread use of immunosuppressive therapies, the growing population of organ transplant recipients, and increased life expectancy [90,91]. Therefore, invasive fungal infections have emerged as a major global clinical concern and are now recognized as infectious diseases associated with substantial morbidity and mortality worldwide [92,93]. Despite this growing disease burden, although vaccines have been successfully developed and clinically implemented for many bacterial infections, no vaccines have been approved for human use against fungal infections to date [94]. Consequently, the prevention and treatment of fungal diseases rely predominantly on antifungal chemotherapy. However, this therapeutic approach is increasingly challenged by the emergence and spread of multidrug-resistant fungal pathogens, in addition to issues related to drug toxicity, limited efficacy, and a rising risk of treatment failure [95,96].

In this context, there is a growing need for novel preventive strategies that complement or potentially replace antifungal drug-based therapies. Fungal EVs have recently attracted attention as promising vaccine candidates, as they contain diverse fungal antigens and possess intrinsic immunostimulatory properties capable of engaging the host immune system.

#### Fungi-Derived EVs

While BMV refers strictly to bacteria-derived vesicles, fungi also release membrane-bound vesicles, termed fungal extracellular vesicles (fungal EVs), which we include here for their conceptual similarity as immunogenic, self-adjuvanting nanoscale platforms. Fungal EVs have recently emerged as potential immunogenic platforms for vaccine development, although this field remains at an early exploratory stage compared with BMV-based strategies [18,97,98].

In a murine model of systemic candidiasis, the protective efficacy of fungal EVs derived from *Candida albicans* was evaluated following intraperitoneal immunization. Mice were immunized once weekly for four consecutive weeks with *C. albicans*-derived EVs in combination with an adjuvant. This immunization regimen elicited robust *C. albicans* antigen-specific IgM and IgG antibody responses. Upon intraperitoneal challenge with a lethal dose of *C. albicans*, vaccinated mice exhibited a marked reduction in fungal burden in key target organs, including the kidney, spleen, and liver. Notably, EVs administered with an adjuvant induced higher levels of TNF-α, IFN-γ, and IL-6 than EVs alone, indicating enhanced immune activation. These findings demonstrate that *C. albicans*-derived EVs can function as immunogenic carriers capable of inducing protective systemic immune responses against disseminated fungal infection [18].

Similarly, the vaccine potential of fungal EVs was evaluated using *Cryptococcus neoformans*-derived EVs. In this study, EVs isolated from a capsule-deficient (cap59-depleted) strain, in which disruption of the CAP59 gene impairs capsular polysaccharide synthesis and consequently reduces capsule-mediated immune evasion, were immunized intraperitoneally to BALB/c mice at 15-day intervals for a total of three doses. EV-specific antibody responses were confirmed by Western blot analysis, and subsequent fungal challenge experiments demonstrated protective effects, supporting the concept that fungal EVs can function as antigen-delivery platforms capable of inducing adaptive immune responses [97].

In parallel, immune responses were evaluated following immunization with *Aspergillus fumigatus*-derived EVs to assess their immunogenic potential. Analysis of immune cells recovered from BALF revealed enhanced phagocytic activity and immune activation, which were associated with reduced pulmonary fungal burden and preservation of lung architecture. EV immunization also induced the production of EV-specific IgG antibodies. However, EV immunization alone did not significantly improve post-infection survival. When combined with a subclinical dose of amphotericin B, EV immunization significantly improved survival and recovery, suggesting that fungal EVs may function primarily as immunomodulatory adjuncts rather than fully protective vaccine platforms [98].

To summarize, fungal EVs can induce antigen-specific antibody responses, reduce fungal burden, and modulate host immunity in systemic and mucosal infection models. Although currently limited to preclinical settings, these findings highlight fungal EVs as immunologically active structures and support their further development as vaccine platforms to complement existing antifungal therapies.

## 4. BMV-Based Vaccine Platforms for Viral Infection

### 4.1. Viral Infection

Viral infections are characterized by high transmissibility and have repeatedly caused widespread outbreaks and large-scale pandemics, constituting a persistent public health threat throughout human history [99,100]. Owing to these characteristics, vaccination has emerged as a central and effective strategy for the prevention and control of viral infections [100]. Traditionally, viral vaccines were developed primarily as inactivated or live-attenuated formulations; however, recent advances in immunology and biotechnology have led to the rapid diversification of vaccine platforms. These include subunit vaccines based on purified antigens, as well as nucleic acid-based approaches, such as mRNA vaccines, which directly deliver genetic information encoding viral antigens [101].

However, despite these technological advances, accumulating evidence suggests that current vaccines may fail to confer adequate protection because of antigenic variation-driven immune escape and insufficient induction of effective cellular immunity. To address these limitations, BMVs have emerged as attractive platforms for viral vaccine development. These bacteria-derived lipid bilayer vesicles possess inherent immunostimulatory capacity and can be genetically engineered to present non-native antigens, such as viral antigens, on their surface, thereby combining antigen delivery with immune activation [21,102,103].

#### 4.1.1. Influenza Virus Vaccines

**Figure 3:**
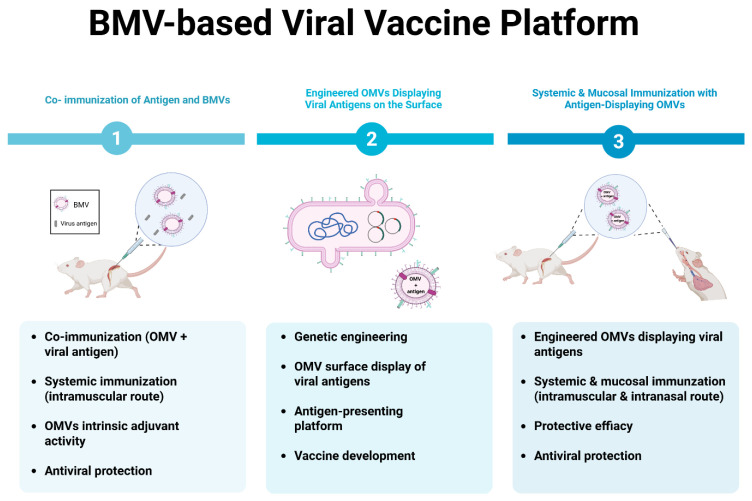
BMV-based viral vaccine platforms. BMVs can be applied as viral vaccine platforms through three strategies: (1) co-immunization of BMVs with viral antigens, using intrinsic adjuvant activity; (2) genetic engineering of BMVs to display viral antigens on their surface as an antigen-presenting platform; and (3) systemic and mucosal immunization using antigen-displaying BMVs to induce protective antiviral immunity. Created with BioRender.com.

Influenza viruses are representative respiratory pathogens characterized by high mutation rates and antigenic drift and shift, which lead to recurrent seasonal outbreaks and frequent evasion of existing vaccine-induced immunity [4,104]. These limitations have driven a sustained demand for novel vaccine platforms capable of improving antigen delivery efficiency while simultaneously eliciting robust innate and adaptive immune responses. In this context, BMVs have emerged as promising alternative platforms for influenza vaccine development [19,22,103,105,106] (Figure 3).

Particularly, OMVs have been shown to function as potent immunological adjuvants capable of amplifying vaccine-induced immune responses. LPS detoxification is a strategy that reduces endotoxin activity by lowering the number of acyl chains on LPS through deletion of lipid A-modifying enzymes or modification of the lipid A structure, thereby attenuating reactogenicity while preserving TLR4-mediated immunostimulation [107]. In a study employing LPS-attenuated *E. coli*-derived OMVs co-immunized with influenza hemagglutinin, OMV-adjuvanted immunization generated antibody titers comparable to those achieved with the conventional alum adjuvant, while more effectively promoting Th1-biased immune responses, as evidenced by increased IgG2c/IgG1 ratios and elevated IFN-γ production [19]. Notably, this OMV-based adjuvant strategy remained effective in high-fat diet-induced obese mouse models, a condition associated with impaired vaccine responsiveness, where it significantly enhanced influenza-specific IFN-γ–secreting T cell responses and TNF-α^+^ CD4^+^ T cell responses. Moreover, OMV-adjuvanted immunization improved survival following homologous viral challenge and conferred partial cross-protection against heterologous influenza strains. Collectively, these findings indicate that OMVs act as effective immunostimulatory adjuvants capable of restoring or enhancing vaccine efficacy even in immunologically compromised host environments [105].

OMVs have further been developed not only as adjuvants but also as antigen presentation platforms, enabling the direct display of viral antigens on the vesicle surface. ClyA, an *E. coli* outer membrane–anchored cytolysin, has been widely used as a representative genetic-fusion-based surface antigen display platform that enables heterologous antigens to be exposed on the OMV outer surface [108]. OMV-based vaccines displaying the conserved influenza A virus antigen M2e on the surface of *E. coli*-derived OMVs via a ClyA fusion protein, which acts as a membrane-associated anchor enabling the display of heterologous antigens on the OMV surface, induced robust Th1-biased immune responses accompanied by high IgG2a antibody titers. Immunized mice exhibited 100% survival following lethal PR8 influenza virus challenge, along with a significant reduction in pulmonary viral loads [103]. Importantly, this platform was validated using detoxified ClearColi-derived OMVs. ClearColi is an engineered *E. coli* host strain in which multiple lipid A biosynthesis genes have been deleted to produce lipid IVa with nearly abolished endotoxin activity, representing a synthetic biology-based approach to OMV detoxification [109]. These detoxified OMVs reproduced antiviral protection across multiple mouse strains and in ferret models, strengthening the safety profile and translational applicability of OMV-based influenza vaccines [106].

A recent study extended the previously established *E. coli*-derived OMV–M2e antigen platform to intranasal immunization to induce both systemic and mucosal immunity. Intranasal immunization resulted in robust antigen-specific IgA production at mucosal sites, together with elevated serum IgG responses. This was accompanied by increased TNF-α^+^ and IL-17^+^ CD4^+^ T cell responses and was associated with a marked reduction in pulmonary viral titers following challenge. Collectively, these findings demonstrate that flu antigen-presenting OMV vaccines effectively integrate antigen delivery with intrinsic immunostimulatory properties to elicit protective immunity at respiratory mucosal surfaces [22].

In summary, BMV-based platforms offer a unique advantage in influenza vaccine development by integrating immunostimulatory activity and efficient antigen delivery within a single nano-scale structure. These properties strongly support the potential of BMVs as next-generation influenza vaccine platforms capable of inducing broader and more durable antiviral protective immunity across diverse immunological contexts.

#### 4.1.2. SARS-CoV-2 Vaccines

SARS-CoV-2 infection is characterized by high transmissibility and the ability to infect ACE2-expressing cells across multiple organs. In severe cases, excessive inflammation can lead to diffuse alveolar damage, cytokine-driven ARDS, and thrombotic complications that contribute to multi-organ damage [110,111]. Owing to these risks, vaccination has been widely adopted as a central preventive strategy to reduce disease severity and mortality associated with SARS-CoV-2 infection [3,112,113]. Although currently approved SARS-CoV-2 vaccines effectively induce systemic antibody responses, their ability to elicit immune responses at the respiratory mucosa, the primary site of viral entry, remains limited [114,115]. In this context, recent studies on BMV-based vaccines have begun to extend conventional systemic immunity-focused approaches by demonstrating the potential to induce mucosal immune responses.

As an initial proof-of-concept study, a vaccine platform based on *N. meningitidis*-derived OMVs mixed with aluminum hydroxide was evaluated for SARS-CoV-2 vaccination. Using a multi-dose vaccination strategy combining intramuscular priming followed by intranasal boosting, this approach induced antigen-specific IgG antibody responses as well as IFN-γ and IL-17A production, demonstrating that OMVs can be utilized as components of SARS-CoV-2 vaccine formulations. This study represents an early attempt to integrate OMVs into conventional adjuvant-based vaccine strategies [20].

Subsequent efforts sought to utilize OMVs as more refined antigen-delivery platforms. In one such study, *E. coli*-derived OMVs were engineered to display short peptide fragments of the SARS-CoV-2 receptor-binding motif (RBM), a Spike subdomain that directly interacts with the host receptor ACE2. To enable surface display, the RBM peptide was fused to FhuD2, a *S. aureus*-derived lipoprotein carrier that anchors the antigen to the OMV outer membrane via its N-terminal lipid moiety, representing a lipoprotein anchor-based surface antigen display strategy [116]. Intraperitoneal immunization with these RBM-displaying OMVs, formulated with aluminum hydroxide, induced high levels of antigen-specific IgG antibodies. Protective efficacy was further supported by reduced viral RNA levels and decreased infectious viral titers (TCID) following viral challenges. Notably, antibodies induced by the RBM antigen also exhibited cross-reactive neutralization against heterologous variants, highlighting the flexibility and scalability of OMV-based platforms for antigen presentation [117].

Meanwhile, studies emphasizing the induction of mucosal immunity, considering the nature of respiratory viral infections, have also been reported. In one such study, *N. meningitidis*-derived OMVs were used to non-covalently associate the SARS-CoV-2 spike protein with the OMV surface via the mCRAMP peptide, an amphipathic cathelicidin peptide that anchors fused antigens onto the OMV surface through its affinity for the negatively charged phosphate groups of LPS lipid A [118]. As a result, intranasal immunization effectively induced not only systemic IgG responses but also robust IgA responses at the respiratory mucosa, and protective efficacy was further demonstrated in a hamster infection model. This study provides clear evidence that OMV-based vaccines can extend beyond systemic immunity to elicit effective mucosal immune responses [119].

In the most recent study, an intranasal vaccine platform based on *Salmonella typhimurium*-derived OMVs conjugated with the SARS-CoV-2 receptor-binding domain (RBD) was presented. Notably, this vaccine induced antigen-specific antibody responses in both serum and BALF following repeated intranasal immunization without the use of additional adjuvants. Protective efficacy was further demonstrated in a hamster infection model, as evidenced by attenuation of body weight loss and a significant reduction in viral burden. This study represents a relatively mature example of an OMV-based SARS-CoV-2 vaccine that integrates the intrinsic immunostimulatory properties of OMVs with a mucosal immunization strategy [21].

Taken together, BMV-based SARS-CoV-2 vaccine studies reported to date have expanded from initial systemic immunity-focused approaches to include strategies aimed at antigen presentation and mucosal immune induction. Collectively, these studies consistently demonstrate that OMVs function both as effective antigen delivery vehicles and as immunostimulatory platforms. However, most reported approaches still rely on multi-dose vaccination strategies, indicating that further studies are required to address immune durability and to optimize vaccination strategies.

#### 4.1.3. Applications for Other Viruses (HCV, HPV, ZIKA, Dengue)

As discussed in Section 4.1.1 and Section 4.1.2, BMV-based vaccine platforms have been primarily investigated in respiratory virus infection models, particularly influenza virus and SARS-CoV-2. However, accumulating evidence indicates that their application is not limited to respiratory viruses. Indeed, BMV-based systems have also been explored in a range of non-respiratory viral infections, including hepatitis viruses as well as Zika virus, human papillomavirus (HPV), and dengue virus. In this section, we briefly review representative studies highlighting the application of BMVs in these non-respiratory viral infection models.

OMV-based adjuvant strategies have also been investigated in the context of hepatitis C virus (HCV) vaccination. In one study, a recombinant fusion protein consisting of truncated HCV core and NS3 regions (rC/N) was mixed with OMVs derived from *N. meningitidis* serogroup B and used to immunize BALB/c mice via the subcutaneous route. The rC/N–OMV mixture elicited a strong Th1-biased immune response, characterized by elevated IFN-γ and IL-2 production and increased IgG2a antibody levels. In addition, OMV-adjuvanted immunization promoted Th2 and Th17 responses and enhanced cytotoxic activity, as indicated by increased granzyme B secretion. Importantly, *N. meningitidis* OMVs induced more potent Th1-type immune responses than conventional adjuvants such as MF59 or Freund’s adjuvant [120].

In a study targeting Zika virus, a hybrid nanostructured vaccine was developed by fusing *N. meningitidis*-derived OMVs with membrane components derived from ZIKV, resulting in the integration of ZIKV antigens into the OMV. Compared with OMVs alone, this hybrid vaccine exhibited enhanced structural stability and induced robust ZIKV-specific IgG antibody responses accompanied by strong neutralizing activity. Additionally, both Th1- and Th2-associated immune pathways were simultaneously activated, along with increased markers related to immune memory [102].

In the context of HPV, a conserved neutralizing epitope, the L2 protein, was selected as the antigen and genetically displayed on the surface of *E. coli*-derived OMVs as a multi-serotype polytope through fusion with a lipoprotein leader sequence and the *Neisseria* factor H binding protein (fHbp) N-terminal domain as a scaffold, employing the same lipoprotein-based surface display strategy described above. This OMV-based vaccine effectively induced antigen-specific IgG antibody responses in mouse immunization studies and exhibited strong cross-neutralizing activity against multiple HPV serotypes. These findings indicate that OMV-based antigen display strategies can elicit broadly protective immune responses against viruses with high serotype diversity, such as HPV [121].

For the dengue virus, envelope domain III (EDIII), a key neutralizing antigen conserved across all four dengue virus serotypes, was selected as the target antigen and genetically displayed on the surface of *E. coli*-derived OMVs through C-terminal ClyA fusion, the same surface display strategy described above, to generate an engineered OMV-based vaccine. Compared with immunization using the antigen alone, this vaccine induced markedly higher levels of EDIII-specific IgG antibodies, along with increased CD4^+^ and CD8^+^ T cell responses expressing Th1-associated cytokines. Furthermore, neutralization assays demonstrated balanced neutralizing activity against all four dengue virus serotypes [122].

Overall, these studies demonstrate that BMV-based vaccine platforms are capable of inducing effective immune responses not only against respiratory viruses but also against a broad range of non-respiratory viruses, including hepatitis viruses, Zika virus, HPV, and dengue virus. These findings highlight the potential of BMV systems to be extended as versatile platforms for antigen delivery and immune stimulation across diverse viral infections.

## 5. Outlook for BMV-Based Vaccines

BMVs are nanoscale vesicles derived from bacterial membranes that simultaneously contain diverse antigenic components and PAMPs. Owing to these features, BMVs can function not only as passive antigen carriers but also as self-adjuvanting vaccine platforms capable of directly activating innate immune responses. Accumulating evidence indicates that BMVs are efficiently internalized by dendritic cells and macrophages, thereby facilitating antigen processing and presentation and inducing diverse adaptive immune responses, including Th1- and Th17-biased T cell responses as well as antibody-mediated immunity. Based on these immunological properties, BMV-based vaccines were initially investigated primarily in models of Gram-negative bacterial infection but have subsequently been extended to Gram-positive bacterial and fungal pathogens. More recently, the application of BMVs in combination with viral antigens has further expanded their utility to viral infection models.

Beyond the self-adjuvanting property described above, BMVs offer several distinctive features as a vaccine platform. Their high amenability to genetic engineering enables defined surface display or luminal encapsulation of heterologous antigens, including those derived from non-bacterial pathogens. Furthermore, BMVs can elicit both systemic and mucosal immune responses depending on the route of administration, making them well-suited for the development of vaccines against pathogens that are transmitted through respiratory or gastrointestinal mucosal surfaces.

However, several challenges remain to be addressed for the clinical application of BMVs. Because BMVs are derived from living bacteria, unavoidable batch-to-batch variability can arise, posing major constraints on large-scale manufacturing and quality control. Particularly, OMVs derived from Gram-negative bacteria may raise clinical safety concerns due to the presence of endotoxin components such as LPS. Therefore, standardized manufacturing, genetic detoxification strategies, and advanced purification and quality control are essential for the clinical application of BMV-based vaccines. Beyond these manufacturing-related issues, the barriers to clinical translation also include the limited availability of large-scale clinical efficacy data: with the notable exception of *Neisseria meningitidis* serogroup B OMV-based vaccines (e.g., 4CMenB/Bexsero), which have been licensed and incorporated into national immunization programs in several countries, providing clinical proof-of-concept that the OMV platform is viable for human use. Most BMV vaccine candidates remain in preclinical or early-phase development, with limited Phase II/III data available. In addition, the development of mucosal BMV vaccines faces specific challenges, as standards for optimal dose, formulation, and stability under mucosal delivery have not yet been established. Although mucosal BMV vaccines offer the advantage of directly inducing mucosal immunity, careful evaluation of potential pulmonary inflammation, airway hyperresponsiveness, mucosal barrier disruption, and local toxicity associated with repeated administration is also warranted. To overcome these limitations and maximize the immunological potential of BMVs, future studies should focus on the refinement of vaccine design strategies.

In the field of viral vaccines, the development of BMV-based vaccine platforms is expected to move beyond conventional strategies based on simple antigen–BMV mixtures or surface antigen display toward more sophisticated, design-driven approaches. Particularly, the production of standardized, internal-empty BMVs followed by the encapsulation of protein antigens or mRNA represents a promising next-generation BMV vaccine strategy. This approach may physically protect the antigen from degradation while enabling the simultaneous delivery of antigens and PAMPs within the same particle, thereby effectively coupling antigen-presenting cell activation with antigen processing and presentation. Notably, mRNA encapsulation strategies may enhance access to the MHC class I pathway via cytosolic translation, thereby supporting the induction of CD8^+^ T cell responses.

For encapsulation-based strategies to provide measurable immunological benefits, several technical challenges remain to be addressed, including antigen loading efficiency and uniformity, as well as effective endosomal escape following intracellular delivery. Nevertheless, BMVs have shown encouraging protective effects in a range of preclinical infection models, and with further optimization of manufacturing processes, safety profiles, and vaccine design, they may serve as adaptable platforms for the development of vaccines against bacteria, fungi and viruses.

## Figures and Tables

**Table 1:** Comparative summary of OMV- and EV-based vaccine studies in bacterial infection models.

Category	Vesicle Source/Pathogen	Experimental Model	Immunization Route	Main Immune Response	Protective Outcomes	Key Interpretation	Refs.
OMV systemic	Gram-negative OMVs from *Klebsiella pneumoniae*, *Acinetobacter baumannii*, *Escherichia coli*, APEC, *Salmonella Enteritidis*	Murine lethal challenge/sepsis models; chicken (poultry) model for APEC and *S.* Enteritidis	Intramuscular or intraperitoneal	Antigen-specific serum IgG/IgM, Th1/Th17-biased cellular immunity, IFN-γ and IL-17 production, dendritic cell activation	Increased survival, reduced bacterial burden in systemic organs such as spleen and liver, reduced systemic inflammation	Gram-negative OMVs function as strong systemic vaccine platforms by combining antigen delivery with intrinsic innate immune stimulation	[16,66,77,78,79]
OMV mucosal	Gram-negative OMVs from *A. baumannii*, *Bordetella pertussis*, *Helicobacter pylori*	Murine airway colonization model; murine respiratory infection model; murine gastric infection model	Intranasal or oral	Secretory IgA production, mucosal antigen-specific IgA, Th17 responses, Th2-biased responses in some gastrointestinal models, local T cell activation	Reduced airway bacterial burden, reduced nasal/lung colonization, alleviated body weight loss, reduced gastric bacterial burden and urease activity	Mucosal OMV immunization can induce localized protective immunity at respiratory and gastrointestinal surfaces, bridging systemic and mucosal immune responses	[59,63,69]
EV systemic	Gram-positive EVs from *Staphylococcus aureus*, *Streptococcus pneumoniae*, *Clostridium perfringens*	Murine pulmonary lethal challenge model; murine lethal challenge model; murine systemic challenge model	Intramuscular or intraperitoneal	EV-specific serum IgG, Th1-dominant cellular immunity, partial Th17 contribution, dendritic cell activation, production of TNF-α, IL-6, IL-12, and G-CSF	Improved survival after lethal challenge, protection against systemic or pulmonary bacterial infection, but variable efficacy depending on bacterial species and strain	Gram-positive EVs can induce meaningful systemic humoral and cellular immunity, with the potential advantage of lacking LPS-associated endotoxin toxicity	[15,86,87]
EV mucosal	Gram-positive EVs from *Streptococcus mutans*; limited evidence from other Gram-positive EV systems	Murine intranasal immunization model	Intranasal, often with mucosal adjuvant formulation	Mucosal IgA induction, systemic IgG responses, possible local T cell activation depending on formulation	Enhanced antigen-specific mucosal IgA responses; protective efficacy remains less established than Gram-negative OMV mucosal vaccines	Gram-positive EV-based mucosal vaccination remains underexplored; current evidence suggests potential for mucosal IgA induction, but effective adjuvant-free mucosal vaccination is still limited	[89]

## Data Availability

No new data were created or analyzed in this study.
